# Heart rate variability in dairy cows with postpartum fever during night phase

**DOI:** 10.1371/journal.pone.0242856

**Published:** 2020-11-25

**Authors:** Takahiro Aoki, Megumi Itoh, Akiko Chiba, Masayoshi Kuwahara, Hirofumi Nogami, Hiroshi Ishizaki, Ken-Ichi Yayou

**Affiliations:** 1 Department of Veterinary Medicine, Obihiro University of Agriculture and Veterinary Medicine, Obihiro, Japan; 2 Department of Veterinary Pathophysiology and Animal Health, Graduate School of Agricultural and Life Sciences, The University of Tokyo, Tokyo, Japan; 3 Faculty of Engineering, Kyushu University, Fukuoka, Japan; 4 Division of Grassland Farming, Institute of Livestock and Grassland Science, National Agriculture and Food Research Organization (NARO), Nasushiobara, Japan; 5 Division of Animal Environment and Waste Management Research, Institute of Livestock and Grassland Science, NARO, Tsukuba, Japan; University of Illinois, UNITED STATES

## Abstract

Autonomic nervous function evaluated by heart rate variability (HRV) and blood characteristics were compared between Holstein Friesian cows that developed postpartum fever (PF; n = 5) and clinically healthy (CH; n = 6) puerperal cows in this case-control study. A cow was defined as having PF when its rectal temperature rose to ≥39.5°C between 1 and 3 days postpartum. We recorded electrocardiograms during this period using a Holter-type electrocardiograph and applied power spectral analysis of HRV. Comparisons between the groups were analyzed by *t* test or Mann-Whitney *U* test, and the relationship between rectal temperature and each parameter was analyzed using multiple regression analysis. Heart rate was higher in PF cows than in CH cows (Mean ± SE, 103.3 ± 2.7 vs. 91.5 ± 1.7 bpm). This result suggested that PF cows had a relatively dominant sympathetic nervous function. Total (44,472 ± 2,301 vs. 55,373 ± 1,997 ms) and low frequency power (24.5 ± 3.8 vs. 39.9 ± 5.3 ms) were lower in PF cows than in CH cows. These findings were possibly caused by a reduction in autonomic nervous function. The total white blood cell count (54.3 ± 5.1 vs. 84.5 ± 6.4 ×10^2^/μL) and the serum magnesium (2.1 ± 0.1 vs. 2.4 ± 0.1 mg/dL) and iron (81.5 ± 8.0 vs. 134.4 ± 9.1 μg/dL) concentrations were lower and the serum amyloid A concentration (277 ± 33 vs. 149 ± 21 μg/mL) was higher in PF cows than in CH cows. These results imply that more inflammation was present in PF cows than in CH cows. Multiple regression analysis showed that both of low frequency power and concentration of serum iron were associated with rectal temperature. We found differences in changes in hematologic results, biochemical findings, and HRV patterns between PF cows and CH cows.

## Introduction

The maintenance of core body temperature is under neuronal control. Fever is defined as a hyperthermic state with an elevated set point. Initiation of the febrile state can occur by a variety of infectious, inflammatory, immunologic, or injurious conditions. In dairy cows, postpartum fever (PF) is predominantly caused by metritis, but mastitis and birth canal injuries can also result in PF. Zhou et al. [1] estimated that 20−40% of postpartum cows are febrile during the first 10 days of the postpartum period, and they reported that early treatment of PF with antibiotics considerably improved rectal temperature, cure rate, and milk yield. Therefore, PF is considered a typical perinatal disease of dairy cows that negatively affects productivity.

The environment of the cows and the human interventions required for herd management can impose physical and/or psychological stress on the cows [2]. In addition to typical examination of stress-related hormone such as cortisol, autonomic nervous function represented by heart rate variability (HRV), have been utilized to assess stress in recent years [3]. The autonomic nervous system (ANS) influences the rate of depolarization of the sinoatrial node of the heart, which is its pacemaker [4]. Changes in the mean heart rate reflect the activity of both the sympathetic and parasympathetic nervous systems, but analysis of HRV permits a more detailed interpretation of ANS activity. HRV parameters of time, frequency, and nonlinear regions can be assessed using ventricular depolarization interval (R–R interval), which reflects the balance between sympathetic and parasympathetic activity, and a more detailed interpretation of autonomic activity can be provided [5]. Stress or painful procedures have been reported to cause a reduction in parasympathetic (vagal) tone, an increase in sympathetic activity, and a reduction in HRV [5]. The parameters that are commonly assessed in HRV analyses include mean heart rate, low frequency power (LF), high frequency power (HF), and the LF/HF ratio. The HF is generally recognized to reflect the modulation of the parasympathetic nervous system, specifically of the vagal nerve [6, 7]. Thus, a reduction in HF values suggests a shift toward sympathetic activity, whereas an increase in HF values suggests a shift toward vagal activity. The LF is thought to be closely associated with variations in peripheral vasomotor tone, reflecting a 10-sec cycle of blood pressure (also known as the Mayer wave) [8, 9]. LF may be used as a stress indicator in dairy cows [10], but because it is affected by both the vagus and sympathetic nerves [6, 11], it has been suggested that is an inadequate marker of sympathetic nerve activity [3, 12, 13]. The LF/HF ratio provides important information regarding the balance of sympathetic and vagus nerves, with higher values being interpreted as shifts toward predominant activation of the sympathetic nervous system [10, 14, 15]. Clinical research in cattle has shown effects of various stressors, such as calving [15–17], heat stress [18], castration [19], dehorning [20, 21], lameness [22], and postoperative pain [23] on HRV. However, to our knowledge, there have been no studies that have evaluated the relationships between perinatal disease and autonomic nervous activity in dairy cows.

Our study objective was to clarify the changes in the balance of ANS in the postpartum dairy cow during the early stage of fever. In our study, cows with a rectal temperature ≥39.5°C within the first 3 days after calving and no other perinatal diseases, such as mastitis or hypocalcemia, were defined as having PF. HRV was recorded and compared with measurements obtained in CH cows. In addition, hematologic and serum biochemical examinations were performed to aid interpretation of the HRV data.

## Materials and methods

**Fig 1:**
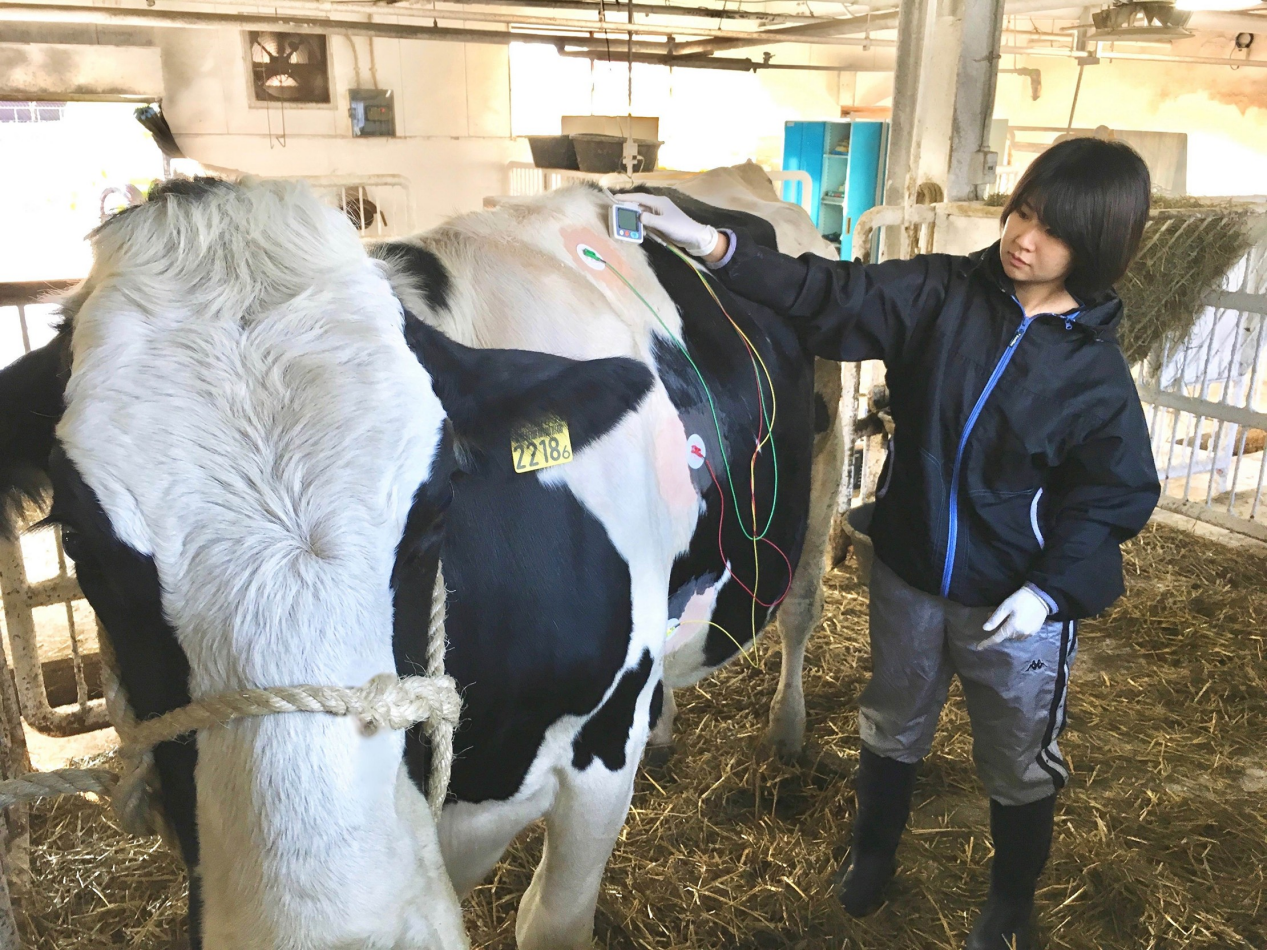
The electrodes are attached to the left side of the chest of the cow after hair removal. The electrocardiographic waveform can be confirmed by monitoring a Holter-type electrocardiograph.

Twenty-two Holstein-Friesian cows that calved at the Field Center of Animal Science and Agriculture, Obihiro University of Agriculture and Veterinary Medicine during October–November 2017, September–November 2018, or August–October 2019 were used in the study. The average predicted 305-day milk yield of the herd was 13,400 kg. The average milk protein and fat content of the herd were 3.47% and 3.97%, respectively. Cows that were close to the expected date of calving were kept at an outside paddock, to monitor regularly for signs of calving, such as a reduction in body temperature, relaxation of the pelvic ligaments, and teat and udder enlargement. Cows that were determined to be approaching parturition were moved to a pen in a barn and observed at regular rounds and using web cameras. Upon delivery, dams were allowed to lick their calves for about an hour, after which they were separated before sucking first colostrum, and the calves were moved to a calf pen that was not visible to the dam. The dams were milked using vacuum pump-type milking equipment and a bucket twice daily, in the morning and evening, and milk yield was recorded. Foremilk was examined routinely via the Californian mastitis test (CMT) using a PL-tester (Zenoaq, Koriyama, Fukushima, Japan). Cows with CMT-positive quarters were excluded from the study and treated by the veterinarians. During the first 24 h after calving (day of calving), dams were left in the calving pen and not sampled. If they exhibited no problem getting up, they were then moved to a tie-stall the day after parturition (1 day postpartum). Sampling was performed between 1 and 3 days postpartum. All protocols and procedures were approved by the Animal Care and Use Committee, Obihiro University of Agriculture and Veterinary Medicine (approval no. 19–44). The individual pictured in **Fig 1** has provided written informed consent (as outlined in PLOS consent form) to publish their image alongside the manuscript. Because the present study did not include any painful procedures, no anesthetics or analgesics were used. After the experiment, the cows were managed in the same way as other milking cows on the farm, and their milk was collected and shipped to the commercial milk company.

Cows with no clinical abnormalities during the study were defined as CH cows (n = 5). Cows with a temperature of ≥39.5°C on 1–3 days postpartum were defined as PF cows (n = 6). A cow that developed hypocalcemia with serum calcium concentration of 3.5 mg/dl was excluded from the data analysis. Even in the absence of clinical signs, cows that experienced dystocia (n = 1) or stillbirth (n = 1) and those who developed retained fetal membranes (n = 3) were excluded from the data analysis of CH cows. In addition, the cow whose electrocardiograph was out of place during the study (n = 5) was excluded from the data analysis.

**Fig 2:**
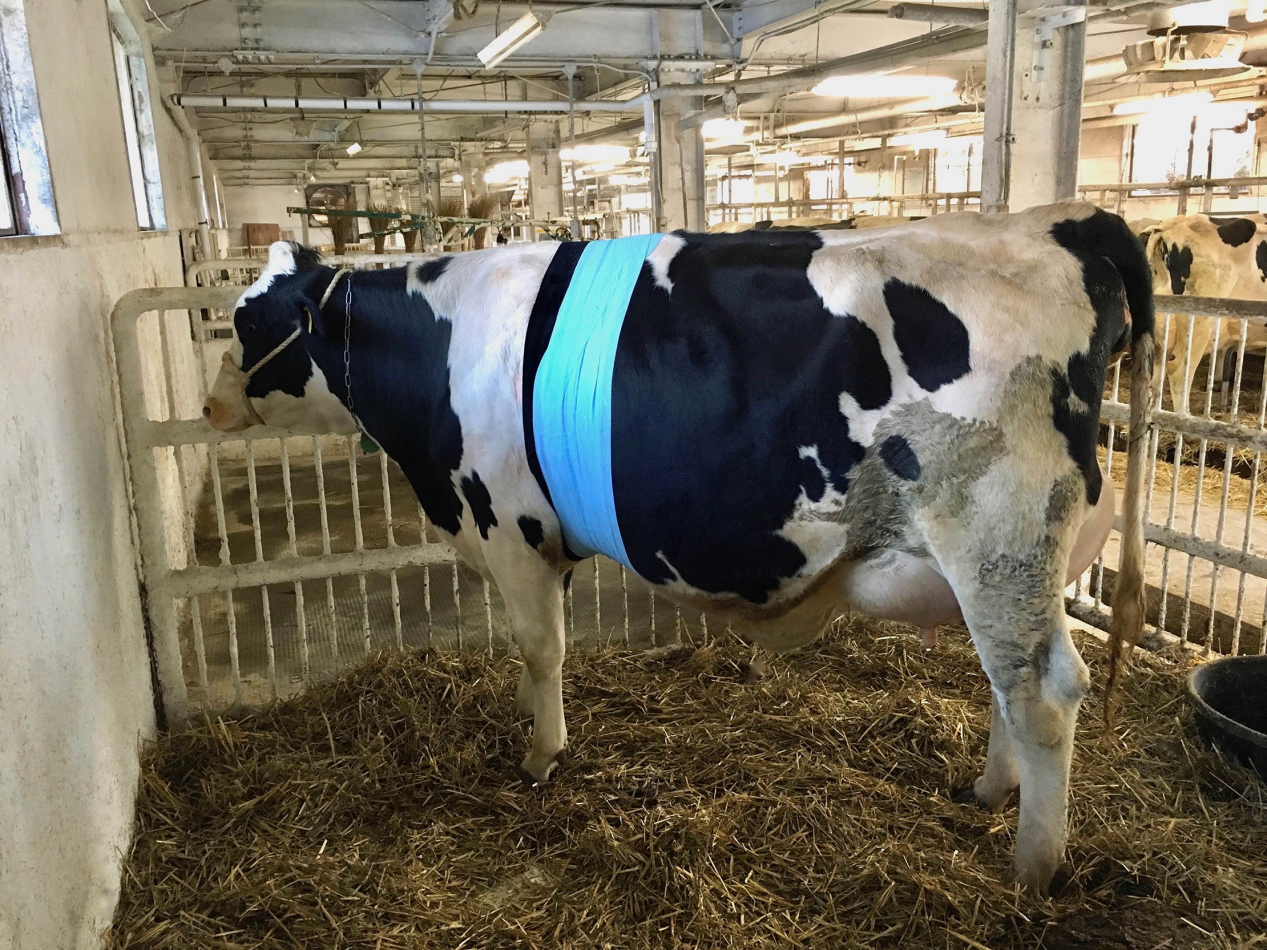
The electrodes, guidance cords, and a Holter-type electrocardiograph are covered with a self-adhesive elastic bandage and a felt belt to permit long-term measurement.

Between 10:00 and 15:00, a general physical examination, including the measurement of rectal temperature, was performed, and 15 ml of peripheral blood was collected into 10 ml vacuum tubes (Venoject II VP-P100K, Terumo Corp., Tokyo, Japan) and 5 ml vacuum tubes containing ethylenediaminetetraacetic acid (EDTA) (Venoject II VP-NA050K, Terumo Corp.) by tail venipuncture using 21 gauge × 1½ inch needles (MN-2138MS, Terumo Corp.). Well-trained veterinarians collected blood samples so as not to stress the cow. The samples containing EDTA were used for complete blood counts. Tubes without EDTA were centrifuged for 15 min at 1,000×g after incubation (37°C, 30 min). Serum was withdrawn and frozen at −30°C for serum amyloid A (SAA), cortisol, and other biochemical analyses at a later date. After blood sampling, electrode pads (Skintact Electrode FS-50, Leonhard Lang GmbH, Innsbruck, Austria) were attached to the left side of the chest of each cow after removing the hair using clippers at three sites: slightly ventral caudal to the withers, immediately caudal to the scapula, and caudal to the elbow (fifth–seventh intercostal spaces); the electrodes were covered with adhesive and electrode fixing pads (Goodtact, Fukuda ME Kogyo Co., Ltd., Tokyo) to avoid dislodgment after the attachment of the guidance cords (Cords for animals, DP-20-3001, Fukuda ME Kogyo Co., Ltd., Tokyo) **(Fig 1)**. The electrodes were also covered with a self-adhesive elastic bandage (PowerFlex Equine 4”, Andover Healthcare, Inc., Salisbury, MA) and a felt belt to permit long-term measurement **(Fig 2)**. Electocardiograms were recorded with baseapex leads using a Holter-type electrocardiograph (SM-50; Fukuda Denshi Co., Ltd., Tokyo, Japan) for about 24 hours.

An automated blood cell counter (Celltac alpha, MEK-6358, Nihon Kohden Corporation, Tokyo, Japan) was used to determine the white blood cell count (WBC), red blood cell count, hemoglobin concentration, hematocrit, and platelet count in EDTA-anticoagulated whole blood. Glucose, lactate, non-esterified fatty acid (NEFA), total cholesterol, triglyceride, beta-hydroxybutyrate, urea nitrogen, creatinine, albumin, aspartate aminotransferase, gamma-glutamyl transferase, calcium, inorganic phosphorus, and magnesium (Mg) and iron (Fe) concentrations were measured in serum using an automated biochemistry analyzer (TBA120FR, Toshiba Medical Systems, Inc., Tochigi, Japan). SAA, an acute inflammatory marker, was measured using commercially available enzyme-linked immunosorbent assay kits (Tridelta Phase Range kit, Tridelta Development Ltd., Wicklow, Ireland). Serum cortisol concentration measurements were outsourced to the Obihiro Clinical Laboratory, Inc. (Obihiro, Japan). The intra- and inter-assay coefficient of variation and the sensitivity range for all of these metabolites are shown in **S1 Table**.

The recorded ECGs were analyzed with an ECG processor analyzing system (SRV-2W, Softron Co., Ltd., Tokyo, Japan) as described previously [24]. The program first detected R waves and calculated the R-R interval tachogram as the raw heart rate variability in sequence order. If there were artifacts in ECGs, these areas were omitted and excluded from the analysis. From this tachogram, data sets of 512 points were resampled at 200 msec. We applied each set of data to the Hamming window and a fast Fourier transform to obtain the power spectrum of the fluctuation. The low frequency (LF) power was set at 0.04–0.1, and the high frequency (HF) power was set at 0.1–1.0 Hz. We set a wider HF power range compared with an earlier study [14], because respiration rate may increase when cows are sick. Heart rate, the LF power, the HF power and the LF/HF ratio were obtained from each recording, and the values were used as indices of autonomic nervous function. Under certain weather conditions, HRV may have a circadian rhythm [25]. Cows reportedly relax at night, and parasympathetic activity predominates. Furthermore, during the present study, people were constantly entering and exiting the cow facility during the day, meaning that the cows may not have been able to relax. Therefore, the mean data values obtained during the period between 22:00 and 04:00, when there was no human entry or exit, were used for intra- and inter-group comparisons.

### Statistical analysis

Multiple regression analysis including the baseline characteristics (i.e., year and month of calving, age, and parity) as explanatory variables was performed to examine the impact on the average rectal temperature during the study. Repeated measures analysis of variance (ANOVA) was performed only in cows in which all data of postpartum days 1−3 were available to examine the within-group variability, the between-groups variability, and interactions. Data for each group at each parameter were examined by Chi-Square goodness-of-fit test to check whether a sample population follows an approximately normal distribution. Differences between the groups were analyzed using Student’s *t* test or Welch’s *t* test when the data followed normal distribution. Differences between the groups were analyzed using the Mann-Whitney *U* test when the data did not follow normal distribution. To determine the magnitude of the impact on rectal temperature during the study, we performed multiple regression analysis using the measurements that differed significantly between the two groups as explanatory variables. All statistical analyses were performed using Excel statistics (Statcel4, Oms Publications, Tokorozawa, Saitama, Japan), and *P* < 0.05 was regarded as statistically significant.

## Results and discussion

The baseline characteristics and rectal temperatures of the CH and PF groups are shown in **Table 1**. Significant effects of year (*F* = 1.33, *P* = 0.29) and month (*F* = 0.80, *P* = 0.41) of calving, age of dams (*F* = 2.45, *P* = 0.17), and parity (*F* = 2.42, *P* = 0.17) on the average rectal temperature during days 1 to 3 postpartum were not observed in the multiple regression analysis.

**Table 1:** Baseline characteristics of cows used in this study and rectal temperature at 1 to 3 days after calving.

Cow No.	Year and month of calving	Age at calving	Parity	Rectal temperature °C		
				Day 1	Day 2	Day 3
**Clinically healthy cows**						
**No. 633**	Sep. 2018	6.8	5	38.8	38.8	39.1
**No. 789**	Oct. 2018	3.0	2	38.9	38.8	38.8
**No. 844**	Oct. 2018	1.8	1	39.3	38.8	38.9
**No. 831**	Nov. 2018	2.1	1	38.7	38.5	39.2
**No. 819**	Sep. 2019	3.3	2	39.1	39.2	39.3
**Cows with PF**^a^						
**No. 742**	Nov. 2017	3.2	2	38.7	39.8	41.0
**No. 837**	Sep. 2018	1.8	1	38.7	39.7	40.2
**No. 838**	Sep. 2018	1.8	1	41.6	41.0	−
**No. 606**	Nov. 2018	7.6	6	−	39.8	40.1
**No. 826**	Aug. 2019	3.0	2	−	39.0	39.7
**No. 845**	Oct. 2019	2.8	2	38.8	39.6	40.5

^a^ PF: Postpartum fever

Repeated measures ANOVA was performed only for cows for whom all data of postpartum days 1−3 were available (**Table 2**). In the HRV parameters, no significance was observed in between-groups variability, within-group variability, or interaction. Statistically significant between-groups variability was observed for white blood cells, serum amyloid A, and iron. There was significant within-group variability for white blood cells, red blood cells, hematocrit, serum amyloid A, lactic acid, total cholesterol, triglyceride, creatinine, and magnesium. Interaction was observed for platelets and serum amyloid A.

**Table 2:** Results of repeated measures ANOVA only for cows with all data of postpartum days 1−3 days (five healthy cows and three with postpartum fever).

	Between groups variability	Within-group variability	Interaction
**Heart rate**	0.09	0.44	0.75
**Total power**	0.10	0.47	0.89
**LF**^**b**^	0.32	0.16	0.10
**HF**^**c**^	0.41	0.44	0.54
**LF/HF**	0.28	0.94	0.97
**White blood cells**	0.03	0.01	0.20
**Red blood cells**	0.68	0.04	0.70
**Hemoglobin**	0.52	0.07	0.90
**Hematocrit**	0.92	0.04	0.86
**Platelets**	0.94	0.22	< 0.01
**Cortisol**	0.64	0.29	0.48
**Serum amyloid A**	0.01	< 0.01	< 0.01
**Glucose**	0.58	0.64	0.20
**Lactic acid**	0.97	0.04	0.56
**Non-esterified fatty acid**	0.26	0.13	0.14
**Total cholesterol**	0.68	< 0.01	0.56
**Triglyceride**	0.45	0.03	0.94
**Urea nitrogen**	0.34	0.14	0.06
**Creatinine**	0.39	0.02	0.07
**Albumin**	0.76	0.33	0.17
**Aspartate aminotransferase**	0.66	0.48	0.69
**γ-glutamyltransferase**	0.53	0.64	0.17
**Calcium**	0.26	0.13	0.50
**Inorganic phosphorus**	0.44	0.68	0.79
**Magnesium**	0.05	< 0.01	0.77
**Iron**	0.01	0.46	0.50

The results of the analysis of the HRV parameters between 22:00 and 04:00 are shown in **Table 3**.

**Table 3:** Heart rate variability parameters collected from 10:00 p.m. to 4:00 a.m. in cows 1–3 days after calving. The results are shown as mean ± standard error. Only results of LF and LF/HF ratio were shown as median value (25−75 percentile) because a sample population does not follow normal distribution.

	Clinically healthy cows	Cows with PF^a^	*P*-value
**Number of cows**	5	6	
**Number of samples**	15	15	
**Heart rate (bpm)**	91.5 ± 1.7	103.3 ± 2.7	<0.01
**Total power (ms)**	55,373 ± 1,997	44,472 ± 2,301	<0.01
**LF**^**b**^ **(ms)**	39.9 ± 5.3	24.5 ± 3.8	0.02
**HF**^**c**^ **(ms)**	3.8 (3.0 − 5.4)	3.3 (1.9 − 5.0)	0.15
**LF/HF (ratio)**	12.9 (11.2 − 16.1)	10.4 (7.8 − 11.5)	<0.01

^a^ PF: Postpartum fever

^b^ LF: Low frequency power

^c^ HF: High frequency power

Heart rate was significantly higher in PF cows than in CH cows. High frequency power of PF cows was lower than that of CH cows, although there was no statistical significance. Total and low frequency power were significantly lower in PF cows than in CH cows.

Heart rate is determined by the depolarization frequency of pacemaker cells in the sinoatrial node. The ANS, comprising sympathetic and parasympathetic divisions, exerts a major influence on these pacemaker cells. Sympathetic nerve activity leads to the release of noradrenaline, which binds to β1 receptors on sinoatrial cells, making them more susceptible to depolarization and increasing the heart rate. In contrast, parasympathetic activity causes the release of acetylcholine, which binds to muscarinic receptors on the sinoatrial cells, raising the threshold for depolarization and reducing the heart rate [26]. The mean heart rate of the cows between 22:00 and 04:00 was significantly higher in the PF group than in the CH group, which suggests that the autonomic balance in the PF group had shifted toward predominant sympathetic activation. Actuary HF power showed a numerically lower value in the PF group than in the CH group. These findings were possibly the result of a relatively dominant sympathetic nervous function caused by a decreased parasympathetic nervous function.

The LF/HF ratio was significantly lower in the PF group than in the CH group. In general, LF/HF ratio is believed to be an indicator of sympathetic and parasympathetic balance, and thus a decrease in LF/HF ratio suggests parasympathetic predominance. However, differences in total power of HRV probably influenced this result, which is not consistent with the mean heart rate data. The total power of HRV is the sum of all the HF, LF, and very low frequency power during the 102.4-sec measurement. This value reflects the amount of ANS activity, which is predominantly sympathetic. In the present study, the total power of HRV was significantly lower in the PF group than in the CH group, implying that the overall ANS activity was lower. Because it is heavily influenced by the total power of HRV, it was difficult to accurately evaluate the balance of autonomic nervous function with the LF/HF ratio in this study. To the best of our knowledge, there have been no studies of the relationship between physical condition and total power of HRV in the field of veterinary medicine; however, in human medicine, a reduction in total power of HRV has been observed in critically ill patients, such as in those with sepsis [27–29].

The results of hematologic and serum biochemical examination are shown in **Table 4**. The WBC count and serum Mg and Fe concentrations were significantly lower and the SAA and serum NEFA concentrations were significantly higher in the PF group than in the CH group.

**Table 4:** Hematologic and serum biochemical parameters in cows 1−3 days after calving. The results are shown as mean ± standard error. Only the results of red blood cells, hemoglobin, glucose, lactic acid, total cholesterol, aspartate aminotransferase, and γ-glutamyltransferase are shown as median value (25−75 percentile) because a sample population does not follow normal distribution.

	Healthy cows	Cows with PF^a^	*P*-value
**Number of cows**	5	6	
**Number of samples**	15	15	
**White blood cells (10**^**2**^**/μL)**	84.5 ± 6.4	54.3 ± 5.1	<0.01
**Red blood cells (10**^**4**^**/μL)**	599 (574 − 650)	621 (598 − 637)	0.71
**Hemoglobin (g/dL)**	10 (9.2 − 11.5)	9.6 (9.2 − 10.6)	0.90
**Hematocrit (%)**	32.8 ± 0.6	32.8 ± 0.7	0.96
**Platelets (10**^**4**^**/μL)**	33.6 ± 2.5	35.5 ± 2.3	0.56
**Cortisol (μg/dL)**	8.2 ± 1.2	11.1 ± 1.8	0.19
**Serum amyloid A (μg/mL)**	149 ± 21	277 ± 33	<0.01
**Glucose (mg/dL)**	64 (53 − 74)	59 (56 − 65)	0.53
**Lactic acid (mg/dL)**	13.2 (9 − 14.1)	12.3 (8.8 − 15.7)	0.88
**Non-esterified fatty acid (μmol/L)**	389 ± 53	578 ± 72	0.04
**Total cholesterol (mg/dL)**	79 (70 − 86)	64 (59 − 84)	0.25
**Triglyceride (mg/dL)**	2.3 ± 0.5	1.7 ± 0.3	0.27
**Urea nitrogen (mg/dL)**	11.8 ± 0.9	10.7 ± 0.8	0.36
**Creatinine (mg/dL)**	0.91 ± 0.03	0.96 ± 0.06	0.48
**Albumin (g/dL)**	3.4 ± 0.1	3.3 ± 0.1	0.49
**Aspartate aminotransferase (IU/L)**	85 (81 − 97)	87 (73 − 105)	0.81
**γ-glutamyltransferase (IU/L)**	20 (16 − 24)	15 (14 − 21)	0.17
**Calcium (mg/dL)**	8.5 ± 0.2	8.2 ± 0.2	0.20
**Inorganic phosphorus (mg/dL)**	5.2 ± 0.3	4.6 ± 0.3	0.16
**Magnesium (mg/dL)**	2.4 ± 0.1	2.1 ± 0.1	<0.01
**Iron (μg/dL)**	134.4 ± 9.1	81.5 ± 8.0	<0.01

^a^ PF: Postpartum fever

SAA is a marker of acute inflammation, which was significantly higher in the PF group than in the CH group. The SAA concentration has been reported to be low (mean 10–20 μg/mL) in CH cows [30–32] and high (mean ≥200 μg/mL) in cows with inflammatory diseases, such as acute mastitis [33]. Compared with previous studies of healthy cattle, CH cows in the present study had higher SAA concentrations (mean 149 μg/mL). This was likely owing to the physiological inflammation that occurs in the postpartum uterus. However, the SAA was significantly higher in the PF group (mean 307 μg/mL) than in the CH group, suggesting that the inflammation in cows affected by PF is more severe than the physiological inflammation that occurs in CH postpartum cows. In human medicine, a reduction in WBC count, accompanied with fever, is one of the diagnostic criteria for systemic inflammatory response syndrome [34]. The reduction in WBC count in peripheral blood results from the rate of migration of WBCs to sites of local inflammation exceeding the rate of their replenishment from the bone marrow or because of adhesion of WBCs to the vascular endothelium, which is promoted by lipopolysaccharides (LPS) from gram-negative bacteria [35]. It was also reported that experimentally induced leukopenia was an acute response that occurs within approximately 12 hours after administration of LPS, and recovered quickly thereafter [36]. In the present study, the WBC count in the PF group was significantly lower than that of the CH group, which is consistent with a stronger inflammatory response in the PF group.

Systemic inflammatory responses in animals induced by experimental doses of LPS have been reported to reduce serum Mg in horses [37] and serum Fe in cattle [38]. In the present study, the serum Mg and Fe concentrations in the PF group were lower than those in the CH group. Further studies, including bacteriological assessments, may be necessary to determine whether such changes in mineral concentrations are the result of an inflammatory response associated with higher circulating LPS concentrations.

Serum NEFA concentration was significantly higher in the PF group than in the CH group. A high NEFA concentration is generally interpreted as reflecting body fat mobilization, which occurs during fasting and starvation [39, 40]. However, in the present study, none of the animals with a fever showed systemic signs, such as anorexia. The ANS, particularly sympathetic nerve activity, has been reported to be involved in body fat mobilization [41]. Therefore, the differences in NEFA concentrations in the present study may have been a consequence of the differences in ANS balance.

**Fig 3:**
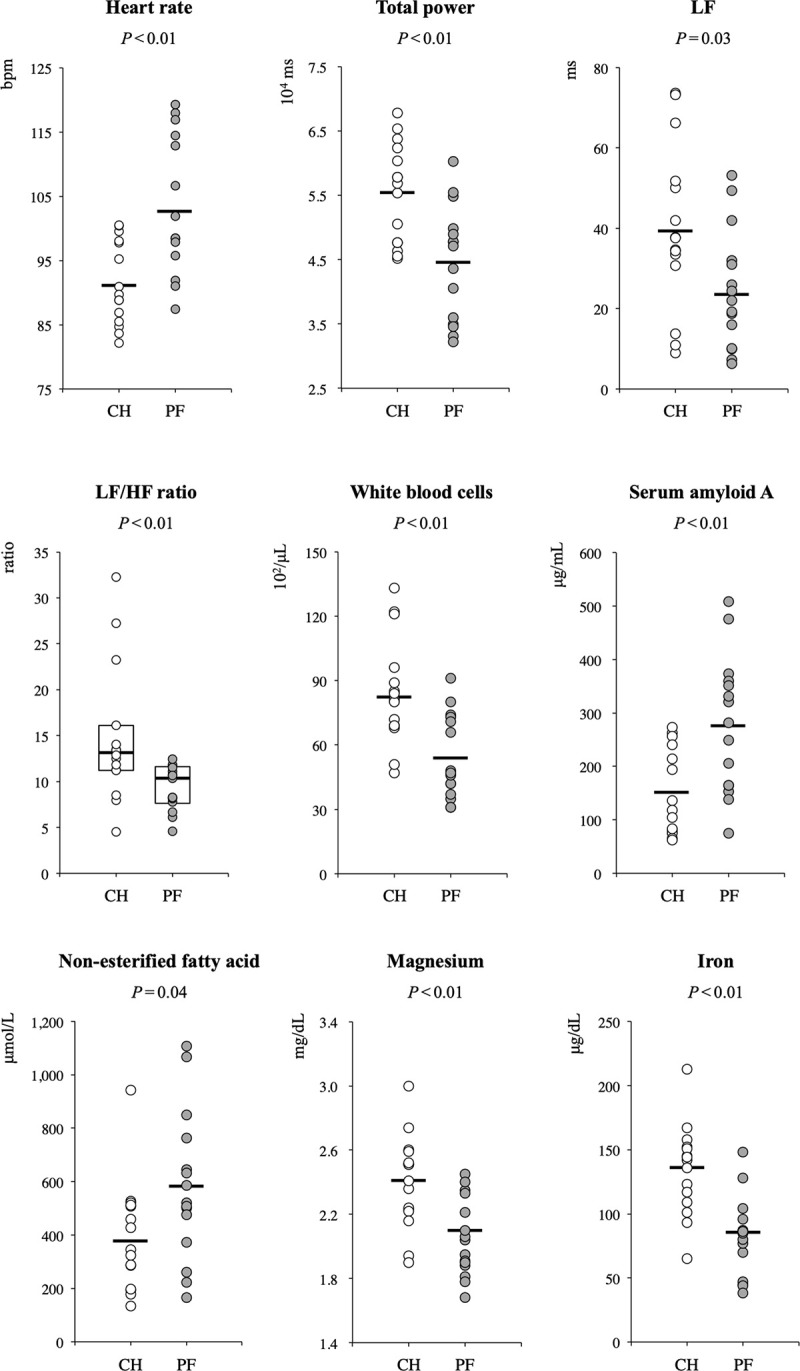
a–c. Dot plots of measuring items with significant differences between the two groups. White circles represent the values of clinically healthy cows (CH; n = 15), and grey circles are of cows that developed postpartum fever (PF; n = 15). The *crossbar* indicates the average value of each group. In the figure of LF/HF ratio, the *box* indicates 25/75 percentiles, and the *center* line of the box indicates the median value. LF, Low frequency power; HF, High frequency power.

Dot plots of measuring items with significant differences between the two groups were shown in **Fig 3A–3C**. We also performed multiple regression analysis using the measurements that differed significantly between the two groups as explanatory variables to determine the magnitude of the impact on rectal temperature during the study. Multiple regression analysis showed that LF and serum iron had significant effects on rectal temperature at 1 to 3 days after calving (**Table 5**).

**Table 5:** Factors affecting rectal temperature at 1 to 3 days after calving.

Explanatory variables	*F*-value	*P*-value	Standardized partial regression coefficient	Partial correlation coefficient
**LF** ^**a**^	9.51	<0.01	0.70	0.57
**Iron**	5.39	0.03	-0.40	-0.46
**White blood cells**	3.66	0.07	-0.51	-0.39
**Serum amyloid A**	0.66	0.43	-0.28	-0.18
**Total power**	0.44	0.52	-0.89	-0.15
**Non-esterified fatty acid**	0.43	0.52	0.12	0.14
**Magnesium**	0.30	0.59	-0.09	-0.12
**Heart rate**	0.04	0.85	-0.24	-0.04
**LF/HF** ^**b**^	0.01	0.92	-0.02	-0.02

^a^ LF: Low frequency power

^b^ HF: High frequency power

In the present study, cows with PF that occurred 1–3 days after calving were compared with CH cattle to evaluate the early stages of the disease. Thus, whether similar results would be obtained for subsequent sub-acute or chronic febrile diseases remains unclear. Moreover, the interpretation of results may have been complicated by the presence of other perinatal diseases. Not only fever detection but also the combination with other findings will be needed to accurately diagnose certain diseases, such as APM.

A limitation of this study was that the sample size was too small to perform sufficient epidemiologic analysis. The cow whose electrocardiograph was out of place during the study was excluded from the data analysis. Improving the mounting method of the electrocardiograph is necessary to obtain the data more stably.

## Conclusions

ANS activation was analyzed using heart rate, HRV, and blood parameters in cows with PF, and changes different from those of CH cows were observed. This could provide useful basic information for the development of wearable health management devices to monitor biological signals.

## Supporting information

S1 Table The intra- and inter-assay coefficient of variation (CV), and measuring range of each item in hematologic and serum biochemical analysis. (XLSX)

S2 Table Heart rate variability, hematologic and serum biochemical parameters in cows 1–3 days after calving. (XLSX)

## References

[1] Zhou C, Boucher JF, Dame KJ, Moreira M, Graham R, Nantel J et al Multilocation trial of ceftiofur for treatment of postpartum cows with fever. J Am Vet Med Assoc. 2001;219: 805−808. 10.2460/javma.2001.219.805 11561658

[2] Carroll JA, Forsberg NE. Influence of stress and nutrition on cattle immunity. Vet Clin North Am Food Anim Pract. 2007;23: 105–149. 10.1016/j.cvfa.2007.01.003 17382844

[3] Mohr E, Langbein J, Nürnberg G. Heart rate variability: a noninvasive approach to measure stress in calves and cows. Physiol Behav. 2002;75: 251–259. 10.1016/s0031-9384(01)00651-5 11890975

[4] Goldberger JJ. Sympathovagal balance: how should we measure it? Am J Physiol. 1999;276: 1273–1280. 10.1152/ajpheart.1999.276.4.H1273 10199852

[5] Porges SW. Cardiac vagal tone: a physiological index of stress. Neurosci Biobehav Rev. 1995;19: 225–233. 10.1016/0149-7634(94)00066-a 7630578

[6] Akselrod S, Gordon D, Madwed JB, Snidman NC, Shannon DC, Cohen RJ. Hemodynamic regulation: investigation by spectral analysis. Am J Physiol. 1985;249: 867–875. 10.1152/ajpheart.1985.249.4.H867 4051021

[7] Malliani A. Association of heart rate variability components with physiological regulatory mechanisms In: Malik M, Camm AJ, editors. Heart rate variability. New York: Futura Publishing; 1995 pp. 173–178.

[8] Berntson GG, Bigger TJ, Eckberg DL, Grossman P, Kaufmann PG, Malik M, et al Heart rate variability: origins, methods, and interpretive caveats. Psychophysiology. 1997;34: 623–648. 10.1111/j.1469-8986.1997.tb02140.x 9401419

[9] Hamner JW, Morin RJ, Rudolph JL, Taylor JA. Inconsistent link between low-frequency oscillations: R−R interval responses to augmented Mayer waves. J Appl Physiol 2001;90: 1559–1564. 10.1152/jappl.2001.90.4.1559 11247960

[10] Kovács L, Jurkovich V, Bakony M, Szenci O, Póti P, Tőzsér J. Welfare implication of measuring heart rate and heart rate variability in dairy cattle: literature review and conclusions for future research. Animal. 2014;8: 316–330. 10.1017/S1751731113002140 24308850

[11] Houle MS, Billman GE. Low-frequency component of the heart rate variability spectrum: a poor marker of sympathetic activity. Am J Physiol 1994;276: 215–223. 10.1152/ajpheart.1999.276.1.H215 9887035

[12] Després G, Veissier I, Boissy A. Effect of autonomic blockers on heart period variability in calves: evaluation of the sympathovagal balance. Physiol Res. 2002;51: 347–353. 12449432

[13] Hagen K, Langbein J, Schmied C, Lexer D, Waiblinger S. Heart rate variability in dairy cows—influences of breed and milking system. Physiol Behav. 2005;85: 195–204. 10.1016/j.physbeh.2005.03.019 15894344

[14] von Borell E, Langbein J, Després G, Hansen S, Leterrier C, Marchant-Forde J, et al Heart rate variability as a measure of autonomic regulation of cardiac activity for assessing stress and welfare in farm animals—a review. Physiol Behav. 2007;92: 293–316. 10.1016/j.physbeh.2007.01.007 17320122

[15] Kovács L, Tőzsér J, Kézér FL, Ruff F, Aubin-Wodala M, Albert E, et al Heart rate and heart rate variability in multiparous dairy cows with unassisted calvings in the periparturient period. Physiol Behav. 2015;139: 281–289. 10.1016/j.physbeh.2014.11.039 25449409

[16] Kovács L, Kézér FL, Ruff F, Szenci O. Timing of obstetrical assistance affects peripartal cardiac autonomic function and early maternal behavior of dairy cows. Physiol Behav. 2016;165: 202–210. 10.1016/j.physbeh.2016.08.001 27494992

[17] Nagel C, Trenk L, Aurich C, Ille N, Pichler M, Drillich M, et al Sympathoadrenal balance and physiological stress response in cattle at spontaneous and PGF2α-induced calving. Theriogenology. 2016;85: 979–985. 10.1016/j.theriogenology.2015.11.009 26699278

[18] Bun C, Watanabe Y, Uenoyama Y, Inoue N, Ieda N, Matsuda F, et al Evaluation of heat stress response in crossbred dairy cows under tropical climate by analysis of heart rate variability. J Vet Med Sci. 2018;80: 181–185. 10.1292/jvms.17-0368 29225303 PMC5797879

[19] Pieler D, Peinhopf W, Becher AC, Aurich JE, Rose-Meierhöfer S, Erber R, et al Physiological and behavioral stress parameters in calves in response to partial scrotal resection, orchidectomy, and Burdizzo castration. J Dairy Sci. 2013;96: 6378–6389. 10.3168/jds.2013-6683 23932135

[20] Stewart M, Stafford KJ, Dowling SK, Schaefer AL, Webster JR. Eye temperature and heart rate variability of calves disbudded with or without local anaesthetic. Physiol Behav. 2008;93: 789–797. 10.1016/j.physbeh.2007.11.044 18177678

[21] Stewart M, Stookey JM, Stafford KJ, Tucker CB, Rogers AR, Dowling SK, et al Effects of local anesthetic and a nonsteroidal antiinflammatory drug on pain responses of dairy calves to hot-iron dehorning. J Dairy Sci. 2009;92: 1512–1519. 10.3168/jds.2008-1578 19307632

[22] Kovács L, Kézér FL, Jurkovich V, Kulcsár-Huszenicza M, Tőzsér J. Heart rate variability as an indicator of chronic stress caused by lameness in dairy cows. PLoS One. 2015;10: e0134792 10.1371/journal.pone.0134792 26270563 PMC4536120

[23] Frondelius L, Hietaoja J, Pastell M, Hänninen L, Anttila P, Mononen J. Influence of postoperative pain and use of NSAID on heart rate variability of dairy cows. J Dairy Res. 2018;85: 27–29. 10.1017/S0022029917000760 29468992

[24] Kuwahara M, Hashimoto S, Ishii K, Yagi Y, Hada T, Hiraga A, et al Assessment of autonomic nervous function by power spectral analysis of heart rate variability in the horse. J Auton Nerv Syst. 1996;60: 43–48. 10.1016/0165-1838(96)00028-8 8884694

[25] Kovács L, Kézér FL, Ruff F, Szenci O. Cardiac autonomic activity has a circadian rhythm in summer but not in winter in non-lactating pregnant dairy cows. Physiol Behav. 2015;155: 56–65. 10.1016/j.physbeh.2015.11.031 26639202

[26] Gordan R, Gwathmey JK, Xie LH. Autonomic and endocrine control of cardiovascular function. World J Cardiol. 2015;7: 204–214. 10.4330/wjc.v7.i4.204 25914789 PMC4404375

[27] Garrard CS, Kontoyannis DA, Piepoli M. Spectral analysis of heart rate variability in the sepsis syndrome. Clinical Autonomic Research 1993;3: 5–13. 10.1007/BF01819137 8386574

[28] Korach M, Sharshar T, Jarrin I, Fouillot JP, Raphaël JC, Gajdos P, et al Cardiac variability in critically ill adults: influence of sepsis. Crit Care Med. 2001;29: 1380–1385. 10.1097/00003246-200107000-00013 11445691

[29] Werdan K, Schmidt H, Ebelt H, Zorn-Pauly K, Koidl B, Hoke RS, et al Impaired regulation of cardiac function in sepsis, SIRS, and MODS. Can J Physiol Pharmacol. 2009;87: 266–274. 10.1139/Y09-012 19370080

[30] Alsemgeest SP, Kalsbeek HC, Wensing T, Koeman JP, van Ederen AM, Gruys E. Concentrations of serum amyloid-A (SAA) and haptoglobin (HP) as parameters of inflammatory diseases in cattle. Vet Q. 1994;16: 21–23. 10.1080/01652176.1994.9694410 8009814

[31] Bagga A, Randhawa SS, Sharma S, Bansal BK. Acute phase response in lame crossbred dairy cattle. Vet World. 2016;9: 1204–1208. 10.14202/vetworld.2016.1204-1208 27956769 PMC5146298

[32] Kaya S, Merhan O, Kacar C, Colak A, Bozukluhan K. Determination of ceruloplasmin, some other acute phase proteins, and biochemical parameters in cows with endometritis. Vet World. 2016;9: 1056–1062. 10.14202/vetworld.2016.1056-1062 27847413 PMC5104712

[33] Nazifi S, Khoshvaghti A, Gheisari HR. Evaluation of serum and milk amyloid A in some inflammatory diseases of cattle. Iranian Journal of Veterinary Research 2008;9: 222–226.

[34] Sibbald WJ, Doig G, Inman KJ. Sepsis, SIRS and infection. Intensive Care Med. 1995;21: 299–301. 10.1007/BF01705407 7650251

[35] Stockham SL, Scott MA. Leukocytes In: Fundamentals of veterinary clinical pathology. 2nd ed. Ames: Blackwell Publishing; 2008 pp. 53–106.

[36] Jacobsen S, Toelboell T, Andersen PH. Dose dependency and individual variability in selected clinical, haematological and blood biochemical responses after systemic lipopolysaccharide challenge in cattle. Vet Res. 2005 Mar-Apr;36(2):167–78. 10.1051/vetres:2004062 15720970

[37] Toribio RE, Kohn CW, Hardy J, Rosol TJ. Alterations in serum parathyroid hormone and electrolyte concentrations and urinary excretion of electrolytes in horses with induced endotoxemia. J Vet Intern Med. 2005;19: 223–231. 10.1892/0891-6640(2005)19&lt;223:aispha&gt;2.0.co;2 15822568

[38] Lohuis JA, Verheijden JH, Burvenich C, van Miert AS. Pathophysiological effects of endotoxins in ruminants. 2. Metabolic aspects. Vet Q. 1988;10: 117–125. 10.1080/01652176.1988.9694158 3046115

[39] Laurell S. Plasma free fatty acids in diabetic acidosis and starvation. Scand J Clin Lab Invest. 1956;8: 81–82. 10.3109/00365515609049249 13337131

[40] Fredrickson DS, Gordon RS Jr. Transport of fatty acids. Physiol Rev. 1958;38: 585–630. 10.1152/physrev.1958.38.4.585 13590931

[41] Havel RJ, Goldfien A. The role of the sympathetic nervous system in the metabolism of free fatty acids. Journal of Lipid Research 1959;1: 102–108.

